# A Versatile Nanocarrier—Cubosomes, Characterization, and Applications

**DOI:** 10.3390/nano12132224

**Published:** 2022-06-29

**Authors:** Cristiana Oliveira, Celso J. O. Ferreira, Miguel Sousa, Juan L. Paris, Ricardo Gaspar, Bruno F. B. Silva, José A. Teixeira, Pedro Ferreira-Santos, Claudia M. Botelho

**Affiliations:** 1 CEB—Centre of Biological Engineering, University of Minho, Campus de Gualtar, 4710-057 Braga, Portugal; pg42888@alunos.uminho.pt (C.O.); celso_jof@hotmail.com (C.J.O.F.); a83628@alunos.uminho.pt (M.S.); jateixeira@deb.uminho.pt (J.A.T.); pedrosantos@ceb.uminho.pt (P.F.-S.); 2 LABBELS—Associate Laboratory, 4710-057 Braga, Portugal; 3 INL—International Iberian Nanotechnology Laboratory, Av. Mestre José Veiga s/n, 4715-330 Braga, Portugal; ricky__g@hotmail.com (R.G.); bruno.silva@inl.int (B.F.B.S.); 4 CF-UM_UP Department of Physics, University of Minho, Campus de Gualtar, 4710-057 Braga, Portugal; 5 Andalusian Centre for Nanomedicine and Biotechnology-BIONAND, 29590 Málaga, Spain; juanluisparis@ucm.es; 6 Allergy Research Group, Instituto de Investigación Biomédica de Málaga—IBIMA, 29590 Málaga, Spain

**Keywords:** nanotechnology, nanomedicine, delivery systems, cubosomes

## Abstract

The impact of nanotechnology on the exponential growth of several research areas, particularly nanomedicine, is undeniable. The ability to deliver active molecules to the desired site could significantly improve the efficiency of medical treatments. One of the nanocarriers developed which has drawn researchers’ attention are cubosomes, which are nanosized dispersions of lipid bicontinuous cubic phases in water, consisting of a lipidic interior and aqueous domains folded in a cubic lattice. They stand out due to their ability to incorporate hydrophobic, hydrophilic, and amphiphilic compounds, their tortuous internal configuration that provides a sustained release, and the capacity to protect and safely deliver molecules. Several approaches can be taken to prepare this structure, as well as different lipids like monoolein or phytantriol. This review paper describes the different methods to prepare nanocarriers. As it is known, the physicochemical properties of nanocarriers are very important, as they influence their pharmacokinetics and their ability to incorporate and deliver active molecules. Therefore, an extensive characterization is essential to obtain the desired effect. As a result, we have extensively described the most common techniques to characterize cubosomes, particularly nanocarriers. The exceptional properties of the cubosomes make them suitable to be used in several applications in the biomedical field, from cancer therapeutics to imaging, which will be described. Taking in consideration the outstanding properties of cubosomes, their application in several research fields is envisaged.

## 1. Introduction

Nanotechnology plays an important role in several research areas. Its impact on the nanomedicine field has been significant. As an example, nanotechnology allowed the development of carriers enhancing drug delivery efficiency, which led to a higher treatment effectiveness. Different authors stated that the combination of nanocarriers with active pharmaceutical ingredients [1,2], resulted in an efficient and safe cancer immunotherapy [3,4]. This new approach significantly reduced the negative effects by enabling the selective accumulation of the toxic drug in the desired area, reducing the dose received by healthy tissues [5]. Furthermore, the use of nanocarriers can also enhance the solubility and stability of some active ingredients [6].

Nanotechnology allows the development of complex nano-systems with high specificity to the target. It is possible to guide a carrier to the desired site through its functionalization, promoting the carrier internalization into cells. This fact allows the delivery of the cargo into subcellular compartments [3,7]. Therefore, the ability to targeted tumor cells increases the anti-tumor action, such as anti-tumor immunity. This can be achieved through the delivery of immunomodulators, which will modify the tumor immune environment, leading to the formation of tumor-specific immune cell subpopulation [3,8]. The possibility of further activating the immune system by enabling the intracellular delivery, through nanocarrier, of tumor-associated antigens and adjuvants to antigen-presenting cells, such as dendritic cells or T cells, macrophages, and B cells, has also been reported [9].

The advances in the nanocarriers field led to the design of nanoparticles with the ability to encapsulate distinct molecules at the same time [10,11]. Another advantage of nanoparticles is their ability to accumulate at the tumor site, releasing the encapsulated molecules in a controlled manner and consequently increasing the drug concentration at the site. This will enhance the therapeutic effect [12]. Moreover, due to the size-exclusion effect, nanoparticles can be resistant to drug efflux, mediated by multidrug resistance transporters. This characteristic enables an effective intracellular concentration of drugs [13].

It is known that particular molecules, like lipids, have the ability to self-assemble, incorporating different active biomolecules. As demonstrated by several authors, the incorporation of bioactive molecules into these structures significantly improved the success rate of several medical treatments [14,15]. This was particularly due to the ability to release the active molecules in a controlled and sustained manner at the desired site [16,17,18,19], with reduced secondary effects.

Among all the lipidic carriers developed over the years, the cubosomic lipid structure has drawn attention. The cubosomes can incorporate molecules with distinct chemical characteristics, apolar, polar, and amphilic molecules simultaneously. This characteristic allows the combination of drugs with different modes of action, creating a synergism [20]. Cubosomes are nanosized dispersions of lipid bicontinuous cubic phases in water, consisting of a lipidic interior and aqueous domains folded in a cubic lattice [20].

Cubosomes’ preparation premise is based on the self-assembly ability of the components into a cubic phase. Cubosomes can be prepared using different molecules, like monoolein (lipid) that, in contact with water, self-assembles into different structures. The versatility of the cubosome platform makes them ideal for use in several applications, from cancer therapy [21] to transfection systems. Thus, in this review paper, the preparation and characterization methods of lipidic nanosystems, mainly cubosomes, and their potential for biomedical applications, will be discussed.

## 2. Lipidic Systems

A drug delivery system’s main purpose is to safely transport a compound in the body until it reaches the targeted biological site. At the site it should elicit an effective therapeutic response. To achieve this purpose, the systems should be designed in order to assure the active molecule bioavailability and pharmacological behavior, with reduced side effects [22].

When developing a drug delivery system, it is important to take in consideration the administration route (oral, nasal, transdermal, intravenous, intramuscular, etc.). A nanocarrier can have distinct structures. It can have a polymeric nanoparticle [23,24], micelles [25], liposomes [26], and cubosomes, the last two being lipid-based systems.

**Figure 1:**
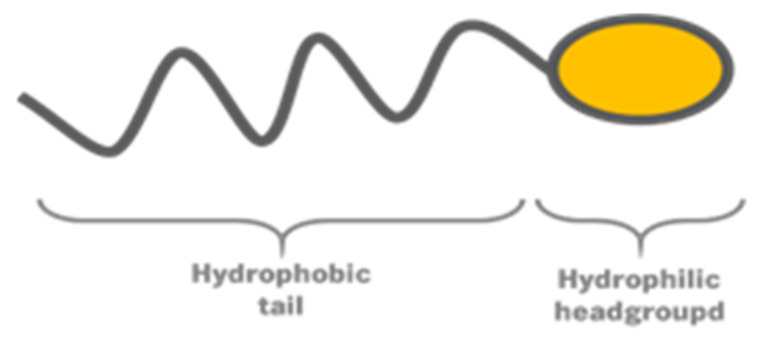
Schematic representation of a polar lipid molecule. Polar lipidic molecules present two domains: a hydrophilic headgroup (polar) and a hydrophobic tail (apolar).

Lipid-based systems are commonly used as drug delivery systems, especially due to its ability to improve solubility and bioavailability of poor water-soluble compounds [27]. The lipid-base systems are formed by lipid molecules, as the name of the system indicates. As illustrated in Figure 1, lipid molecules have two different domains: a hydrophilic headgroup, and a hydrophobic tail composed of one or more hydrocarbon chains. Molecules with such properties are named amphiphilic, and it is this characteristic that leads to self-assembled structures.

### 2.1. Self-Assembly of Lipidic Structures

The self-assembly of lipidic nanoparticles is a consequence of a process called hydrophobic effect. In the presence of water, the amphiphilicity of the lipidic molecules leads to the formation of two opposite forces: one favoring the aggregation of the lipid and the other repelling it. The aggregation force is due to a difference in the polarity between the hydrocarbon chain and water. When these molecules are placed in contact with water, the water molecules surrounding the tail of the lipid form organized networks, decreasing entropy and consequently increasing the system free energy [28,29]. To regain the lost entropy, the adsorbed water molecules need to be released. There are two ways to achieve this goal: either by the adsorption of lipidic molecules to an interface, or by the association of hydrocarbon chains of different molecules, resulting in structures with low overall energy system [28]. These structures are characterized by a hydrophobic core (hydrocarbon chains) and a hydrophilic surface (polar headgroups).

When the tails of different lipidic molecules associate, due to the hydrophobic effect, the hydrophilic headgroups get closer. As a consequence, these polar groups start repelling each other (the repelling force).

**Figure 2:**
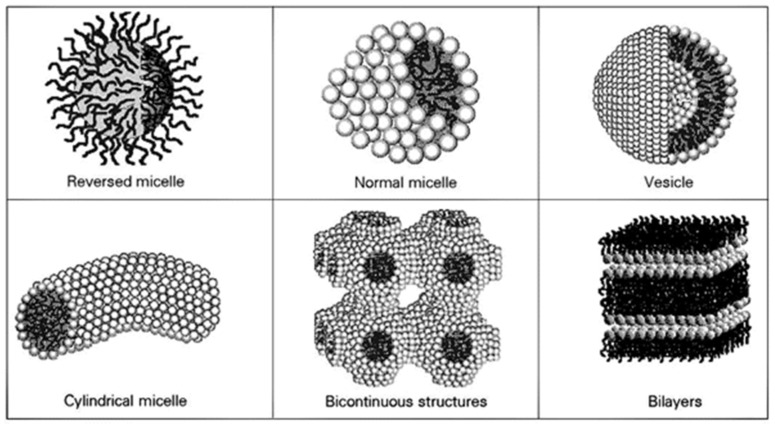
Most common self-assembly structures of amphiphilic molecules, including micelle, vesicle, bilayer, and bicontinuous phases. Reproduced from Florenzano et al., 1997 [30] (work licensed under a Creative Commons Attribution 4.0 International License, 2022).

Several parameters influence the self-assembly of lipidic structures, namely the balance between two opposite forces, the chemical structure of the surfactant, the environment conditions (temperature, pH, pressure, etc.), the total bulk-phase composition, among others [28,29]. As illustrated in Figure 2, some of the most common structures formed are micelles, vesicles, bilayers, and bicontinuous phases [30].

### 2.2. Packing Parameter and Spontaneous Curvature

One way to predict the type of structure that will be formed by a surfactant-water system is the packing parameter, *P_s_*. This parameter is the ratio of hydrophobic volume to hydrophilic projected volume, and can be determined by the following expression previously reported [28] (1):

(1) $$P_{s}=\frac{v_{c}}{a_{0\;}l_{c}}$$

where *v_c_* is the volume of the hydrocarbon chain, *a*_0_ is the area of the headgroup, and *l_c_* is the maximum length of the hydrocarbon chain in the fluid environment. 

The volume can be determined according to Equation (2). Depending on the number of carbons, *n_c_*, the volume can be calculated with:

(2) $$v_{c}=0.0274+0.0269n_{c}\;\left(nm^{3}\right)$$

Similarly, a generic expression can be used for the determination of the maximum length of the hydrocarbon chain. To do that, the following values are assumed: 0.253 nm as the distance between alternate carbon atoms, 0.21 nm as the van der Waals radius of the terminal methyl group, and 0.06 nm as half the bond distance between the first carbon in the core and that bonded to the head group [28]. Considering *n_c_* as the number of core carbon atoms, the following expression is obtained (3):

(3) $$l_{c}=0.15+0.1265n_{c}\;\left(nm\right)$$

**Figure 3:**
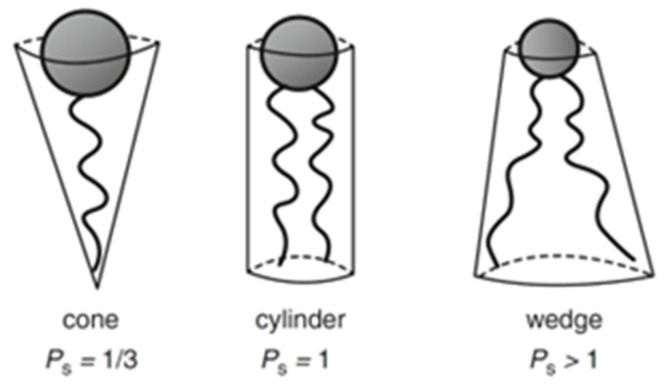
Illustration of the *P_s_* concept and relation with geometrical shapes. Adapted from Marques and Silva, 2013 [31].

The area of the headgroup (*a*_0_) strongly depends on the medium conditions, making its determination more complicated [31]. Once calculated, *P_s_* can assume different values that correspond to different geometric solids (Figure 3). For example, *P_s_* < 0.33 matches a cone shape, *P_s_* = 1 corresponds to a cylinder, and *P_s_* > 1 to an inverted truncated cone.

The expected lipidic structure will be the one that optimizes the packing of the geometric solids described by the *P_s_*. Therefore, if it is a cone shape, the formed structure will be a normal micelle, a hexagonal or cubic phase. If the shape is a cylinder, the structure will correspond to a planar extended bilayer. When the shape is an inverted truncated cone, it means that the volume of the hydrophobic part is significantly bigger than the hydrophilic area of the headgroup, consequently leading to a reversed self-assembled structure [28,32,33].

An alternative way to predict the type of structure obtained by the surfactant-water system is the spontaneous curvature (*H*_0_). The principle behind this concept is equivalent to the one that supports the packing parameter, as they both relate the volumes of the hydrophobic and hydrophilic domains. The difference, however, relies on the considered unit: while the packing parameter uses the molecule as an entity, in the spontaneous curvature, it is the film as a whole that is considered as the unit [31].

The *H*_0_ corresponds to the surface mean curvature when no mechanical stress is being applied. Considering *R_1_* and *R_2_* as two perpendicular radii of curvature, *H*_0_ can be determined by Equation (4):

(4) $$H_{0}=\frac{1}{2}\left(\frac{1}{R_{1}}+\frac{1}{R_{2}}\right)$$

If *H*_0_ > 0, the curvature will be towards the hydrophobic region, and if *H*_0_ < 0, the curvature will be towards the hydrophilic region.

Another important parameter for lipidic system prediction is the Gaussian curvature (*K*), defined according to Equation (5):

(5) $$K={\left(R_{1}R_{2}\right)}^{-1}$$

**Figure 4:**
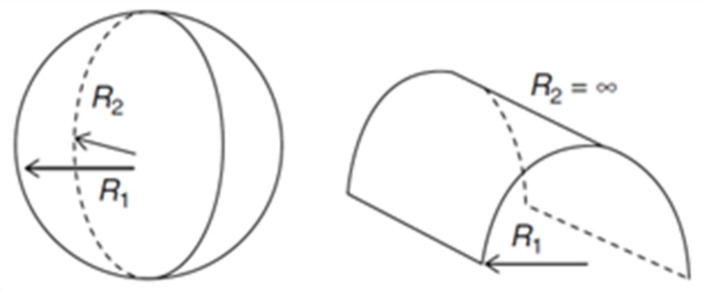
Self-assembled structures radii. Left: sphere with both radii positive; Right: cylinder with a positive finite radius and an infinite radius. Adapted from Marques and Silva, 2013 [31].

It is the relation between H_0_ and K that will allow us to predict the formed structure. Therefore, if *R*_1_ and *R*_2_ have the same finite value, then *K* > 0 ∧ *H* ≠ 0, and the formed structure will be a sphere. If one of the R is finite and the other infinite, *K* = 0 ∧ *H* ≠ 0, and the shape will be a cylinder (Figure 4). In the case of a planar structure there is no curvature, so both *H*_0_ and *K* are zero, thus both R are infinite. However, there are more structures beside the planar that have a *H*_0_ equal to zero, like the bicontinuous cubic phases. In these structures, *R*_1_ and *R*_2_ have the same finite value, but opposite signs, hence *K* < 0 ∧ *H* = 0 [31,34].

### 2.3. Cubosomes

#### 2.3.1. Bicontinuous Cubic Phases

Bicontinuous cubic phases are lyotropic liquid crystalline phases, meaning they possess intermediate properties between solid crystals and isotropic liquids. A unique characteristic of these systems is their ability to remain thermodynamically stable in excess solvent, whereas other systems tend to dissociate [35]. The structure of bicontinuous cubic phases features a single lipidic bilayer that separates two continuous hydrophilic regions that intertwine but never connect. The lipidic surface is contorted into a periodic minimal surface with zero mean surface curvature and negative gaussian curvature [36]. Such a structure confers a high viscosity to the phase, which can limit the pharmaceutical applications [35,36]. One way of overcoming the high viscosity of the bulk phase is the formation of particles. In the presence of excess water, the bulk lipid liquid crystals can be dispersed into colloidal particles that have same properties of the bulk cubic phase, but a lower viscosity [35,36]. These nanoparticles are named cubosomes.

**Figure 5:**
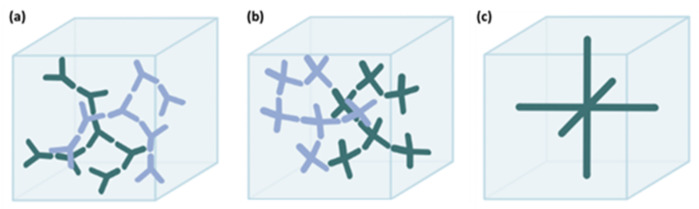
Skeletal graphs of the water channels in the three bicontinuous cubic phases morphologies. (**a**) Ia3d (G-surface), (**b**) Pn3m (D-surface), (**c**) Im3m (P-surface). Adapted from Squires et al., 2006 [40].

Cubosomes are self-assembled lipidic structures with a size that ranges from 50 nm to 500 nm [36] and can be presented in three different morphologies: Ia3d (G-surface), Pn3m (D-surface), and Im3m (P-surface) (Figure 5) [20,37]. In the Ia3d structure, the channels of each hydrophilic region are connected three-by-three; in the Pn3m the pair of water channels is organized on a tetrahedral network (four-by-four); finally, in the Im3m structure, each water channel forms an orthogonal network (six-by-six) [38,39].

Despite previous descriptions of cubic phases in lipid–water systems, cubosomes colloidal preparation and further characterization were performed by Larsson [41] and Landh [42]. The authors referred to them as dispersions of inverted bicontinuous cubic phases where bilayers periodically curved in three dimensions were supporting two separate water networks. Later, Gustafsson et al. was able to corroborate this idea by crossing cryo-TEM images with Small Angle X-ray Scattering reports of these dispersions, which showed that these were in fact cubic liquid crystalline structures [43].

One major advantage of the use of cubosomes over other particles, like liposomes, is their larger hydrophobic region, which enables larger loading capacity of hydrophobic drugs while still enabling loading hydrophilic ones. Indeed, Chang et al. revealed a larger loading of curcumin in phytantriol cubosomes compared to liposomes, and this led to a higher in vitro cytotoxicity of the drug-loaded formulations [44]. Furthermore, due to the cubosomes’ lattice structure, the particle membrane curvature can be tuned independently of its size. This last characteristic is particularly important in the mimicry of highly curved structures, which are characterized for higher membrane to surface area to volume ratio and increased membrane loading capacities [37]. The various features of cubosomes, among which is the possibility for a controlled release and biocompatibility, make cubosomes a great potential delivery system that could allow for better therapeutics with lower dosages.

#### 2.3.2. Principal Constituents

To form cubosomes, three main constituents are required: a lipidic mixture, a stabilizer, and the molecule of interest that will be loaded into the particles. The premise of the formation of cubosomes is based on the self-assembly of these components into a bicontinuous cubic phase.

**Figure 6:**
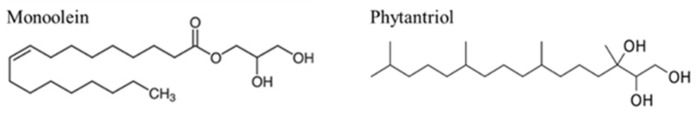
Chemical structure of monoolein and phytantriol.

Starting with the lipidic mixture, the two most used lipids are monoolein and phytantriol (Figure 6). Both lipids form a Pn3m cubic phase morphology, and both are biocompatible and approved for in vivo use [37].

Monoolein, also known as glyceryl monooleate, is a lipidic mixture that consists of glycerides of oleic acid and other fatty acids (mainly monooleate) [20]. This amphiphilic molecule features a hydrocarbon chain attached to the glycerol backbone by an ester bond. Such a bond can occur in any of the three carbon positions of the glycerol backbone. The remaining two carbons have active hydroxyl groups that may form hydrogen bonds with water, making them responsible for giving hydrophilic/polar characteristics to this segment of the monoolein molecule (headgroup). In turn, the hydrocarbon chain (tail) gives the hydrophobic characteristics to monoolein [45,46]. The biocompatibility, biodegradability, non-toxicity, and the ability to form various liquid crystalline structures make monoolein a molecule of great interest [46].

**Figure 7:**
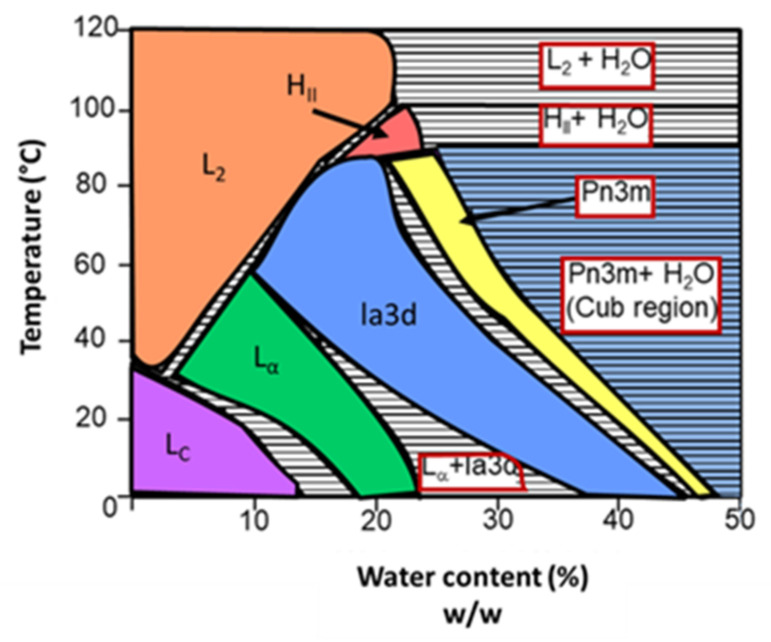
Aqueous phase behavior of the monoolein–water system. L_c_ and L_∝_ represent the lamellar phases (crystalline and fluid, respectively). Ia3d and Pn3m are the inverted bicontinuous cubic phases. L_2_ and H_II_ correspond to the inverted micellar and hexagonal phase, respectively. Adapted from Kulkarni et al., 2011 [46].

When in contact with water, monoolein can originate different self-assembled structures. The aqueous phase behavior of the monoolein–water system is schematized in Figure 7. By analyzing the diagram, it is clear that the various structures that the system can originate depend on the temperature and water content. At low to medium temperatures, an increase of the water content leads to a transition from lamellar phases (first crystalline, then fluid) to inverted bicontinuous cubic phases (Ia3d, followed by Pn3m). Continuing to increase the water content, the cubic phase Pn3m starts coexisting with excess water (for example, at room temperature, approximately 42% of the water content is incorporated onto the phase and the rest is excess). It is in that region that cubosomes are formed (blue on the diagram). At high temperatures, the monoolein–water system forms inverted micellar and hexagonal phases, with or without water excess, depending on the water content [46].

Phytantriol is a biocompatible and well-known lipid, especially in the cosmetic industry [47]. This lipid features a similar phase transition behavior to monoolein, allowing it to gain importance as a viable alternative in cubosomes manufacture. The molecule is composed of a highly branched phytanyl tail (hydrophobic section) and a tri-hydroxy headgroup (hydrophilic section). Unlike monoolein, phytantriol does not feature an ester functional group, thereby it is not susceptible to esterase-catalyzed hydrolysis, positioning phytantriol as a more stable alternative [48,49].

**Figure 8:**
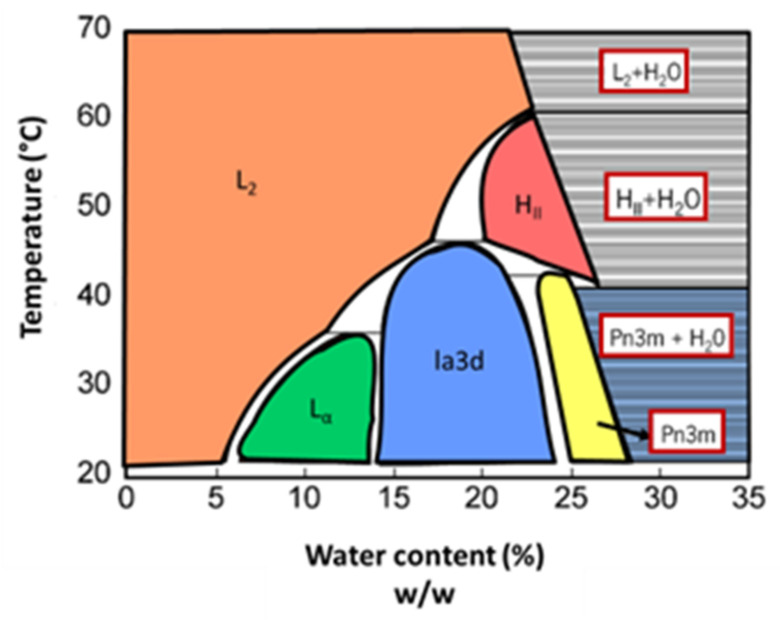
Phase diagram of the phytantriol–water system. L_c_ and L_∝_ represent the lamellar phases (crystalline and fluid, respectively). Ia3d and Pn3m are the inverted bicontinuous cubic phases. L_2_ and H_II_ correspond to the inverted micellar and hexagonal phase, respectively. Adapted from Barauskas and Landh, 2003 [47].

The phase diagram of the phytantriol–water system is shown in Figure 8. At low temperatures and low water content, the formation of a reversed micellar phase is favored. The increase of water content leads to a phase sequence similar to the one of the monoolein–water system: transition to a fluid lamellar phase, followed by the inverted bicontinuous cubic phases Ia3d and Pn3m. In this system, continuing to increase the water content also leads to the coexistence of Pn3m cubic phase and excess water, allowing the creation of cubosomes. At high temperatures, according to the water content, reversed micelle and reversed hexagonal phases are formed [47,48,49,50].

Although bicontinuous cubic phases remain thermodynamically stable in bulk, the same does not happen when the phase is dispersed. From a colloidal perspective, the nanoparticles (i.e., cubosomes) are not stable: the exposure of the hydrophobic domains to external aqueous environment results in a tendency of the particles to aggregate as a way to minimize the free energy of the system [35]. This aggregation, which can occur by different mechanisms, leads to a phase separation between lipid-to-water. In order to avoid this transition, it is necessary to increase the level of energy required for it to happen. One way to achieve this goal is by using stabilizers in the dispersions.

Stabilizers can avoid the aggregation of nanoparticles by coating the outer surface [37]. The coating leads to electrostatic or steric repulsive interactions between molecules, increasing the energy required for the phase separation. In the case of electrostatic interactions, the stability is achieved with polyelectrolytes oppositely charged to the particle interface. The polyelectrolytes adsorb to the particle surface, causing an electrostatic repulsion between particles (Coulomb forces). The repulsive Coulomb forces counterbalance the attractive van der Walls forces, thus avoiding the aggregation [51,52]. One disadvantage of this technique is its sensitivity to the addition of electrolytes, making the steric stabilization a more frequent option [53]. In the steric stabilization, the particles are coated with a non-ionic polymer. When two particles approach each other and the distance between them becomes smaller than twice the thickness of the coating, the polymer chains experience interpenetration and compression. As a result, the entropy in the interaction zones reduces, and a repulsive steric force is originated, stabilizing the particles [54]. In both cases the stabilization provided is limited in time, and the particles eventually tend to aggregate.

The most commonly used stabilizing agents are Pluronics, especially Pluronic F127 (F127). F127 is a water-soluble stabilizer that is composed by three polymer blocks: two polyethylene oxide (PEO) and one polypropylene oxide (PPO), organized in a PEO-PPO-PEO configuration. The PEO has a hydrophilic character, whereas PPO is hydrophobic. This stabilizer acts by adsorbing the hydrophobic block to the surface of the particles, while the hydrophilic blocks form a corona that sterically stabilizes the dispersion [35,55].

It is important to note that stabilizers may interact with the internal structures of the particles. For example, in monoolein cubosomes assembled with a low concentration of F127, the formed structures have a Pn3m morphology. However, if the portion of F127 is excessive, the surface of the nanoparticles becomes saturated, and the polymer may be incorporated within the bilayer. Consequently, the structure transitions from Pn3M to Im3m. In contrast, the same does not happen with phytantriol cubosomes, where high concentrations of F127 do not affect the internal structure [35,55].

Furthermore, it is noteworthy that cubosome cytotoxicity depends on several factors, such as internal nanostructures, lipid chemistry, and the type of stabilizers used [56,57]. A study by Fornasier et al. proved that the cubosomes prepared with polyphosphoester (PPE), structurally analogue of the traditional F127, were significantly less toxic than carriers with F127. This result was observed in the two cell lines evaluated: HEK-293 and HUVEC. It was also demonstrated that the poly(phosphoester)-based formulation has a high hemocompatibility in contrast to cubosomes prepared with F127, which reveals a certain degree of cytotoxicity against erythrocytes [58]. Moreover, several studies reported a half maximal inhibitory concentration (IC50) value of monoolein-based cubosomes in the range of 30–100 µg/mL [56,59,60,61]. Cubosomes are licensed and marketed as a non-parenteral medication in the United Kingdom and are on the US FDA’s list of inactive ingredient guidelines [20,62].

It is worth noting that, even though Pluronic surfactants are the most common stabilizing agents reported in the literature for the preparation of cubosomes, other agents have also been reported, such as D-α-tocopheryl poly(ethylene glycol)1000 succinate [63] and Poloxamer 407 [64].

#### 2.3.3. Cubosome Preparation

For the preparation of cubosomes it is necessary to take into consideration several parameters, such as stability, biocompatibility, and optimum cargo release [35]. Several methods can be used in the preparation of cubosomes, but typically they all follow one of two different approaches: top-down and bottom-up.

Top-down techniques are based on a suitable starting material that then, by the means of high energetic inputs, is sculpted into the desired structures [20]. For the formation of cubosomes, a bulk cubic phase is initially prepared. Afterwards, this starting material is dispersed into an aqueous medium by sonication or high-pressure homogenization, forming the cubosomes [20,37,49]. The chosen lyotropic phases can be obtained by co-melting the desired lipid mixtures in small amounts of water, allowing it to equilibrate [65,66,67]. Another approach is to dissolve the lipid mixture in a hydrotrope for later evaporation [68]. In both cases, it is necessary to add a stabilizer dissolved in water before the dispersion step, whether by sonication or homogenization. Some variations of this process may include heat cycling steps in order to improve dispersity and decrease the number of vesicles formed [66]. Although these are the most common strategies, both present several drawbacks, such as the need for high energetic input, heat generation, and vesicle formation. Moreover, if another solvent is used at the early steps, extra caution is needed. This procedure can lead to increased toxicity, limiting its use for biological applications.

The approaches described lead to reproducible and stable cubosomes [37]. However, top-down approaches have some downsides, such as the formation of vesicles. Vesicles are formed in this context are the result of shear forces that lead to the formation of non-equilibrium particles. These can be reverted to the cubic phase, however it is a long process [65]. Furthermore, it requires a high energetic input to disperse the viscous bulk phase, resulting in sample heating damaging the bioactive molecules incorporated. This fact can impair the translation of this techniques to large scale production [20,37,49].

Bottom-up approaches rely on the initial formation of nanostructures building blocks, that afterwards will assemble into the final structure [20]. An example of a bottom-up approach is the solvent exchange method, also known as solvent shifting and nanoprecipitation. The first step of this technique consists of the preparation of a waterlike precursor solution by dissolving the lipid in a water miscible solvent (e.g., ethanol). Then, the precursor solution is injected in water, resulting in the dilution of the solvent in water, and causing a quick decrease on lipid solubility. Consequently, the lipid self-assembles into a cubosome structure. Additionally, a stabilizer and molecules of interest can be incorporated into the initial solutions [69].

The main advantage of the solvent exchange method over others, especially the top-down approach, is the formation of smaller and more stable particles, with low polydispersity. As this technique does not require a high energetic input, the use of temperature-sensitive molecules is not compromised. However, the need for a water miscible solvent, like ethanol which is cytotoxic, represents a downside of this approach [20,37,69]. The inclusion of another solvent at the final formulation can affect particle structure, for example leading to the formation of vesicles. When Spicer et al. synthesized the first cubosome using the solvent shifting method, it was suggested that the vesicles formed were transitory particles, as precursors of cubosomes [69]. In fact, only upon ethanol evaporation can these vesicles form cubosomes [70].

Recently, solvent exchange has been used in a microfluidic setup for the preparation of monoolein cuboplexes with the cationic phospholid 1,2-dioleoyl-3-trimethylammonium propate (DOTAP) and interfering Ribonucleic Acid. Cuboplexes are cubosomes whose goal is to deliver nucleic acids to cells in order to improve gene silencing efficiency. In 2018, Kim et al. obtained monodisperse cuboplexes using a microfluidic device with a herringbone pattern [71], achieving relatively monodisperse sizes of ca. 200 nm. The particles obtained were able to perform significantly better when compared to the commercially available products. Hence, showing the potential of cubosomes to the medical field while using microfluidics as an alternative method.

Despite the development on cubosomes preparations, there are still a few flaws that need to be overcome. The constant vesicle formation, structural variation, and lack of control over the size can all limit these particle applications. It is important to mention that the use of microfluidics for the preparation of cubosomes overcomes the lack of control size, making microfluidics an interesting approach for the preparation of cubosomes.

#### 2.3.4. Cubosome Characterization

As highlighted previously, cubosomes consist of nanosized lipid-based bicontinuous cubic phases dispersed in excess water and typically stabilized by an amphiphilic polymer. Indifferent to the method chosen to produce them, their physicochemical properties are essential for drug or gene delivery, such as particle size and structure. There are several techniques to characterize the cubosomes. These techniques have been grouped into two categories, direct techniques which provide phase identification, and indirect techniques where measurements lead to phases characterization [72]. In this review, this classification will also be adopted and detailed in the following subsections for the most used techniques for cubosome characterization.

##### 2.3.4.1. Direct Techniques

###### 2.3.4.1.1. Electron Microscopy

Cryogenic transmission electron microscopy (Cryo-TEM) allows the characterization of complex fluid structures, providing a direct visualization of the size and morphology of the nanosystems (in this case, cubosomes). It is also possible to analyze the lattice symmetry and repeated distances [73].

The characteristic “honeycomb” structure of cubosomes has been elegantly imaged by Cryo-TEM in a vast number of studies, using various lipid formulations for diverse applications which include, for example, drug delivery systems [74,75].

Impressive images of dispersed cubic phases were shown by Johnsson et al., where the cubosomes were composed of dioleoylphosphatidylethanolamine (DOPE) supplemented with small amounts of PEGylated GMO [75]. In this particular system, DOPE/PEG(660)-GMO/water, DOPE was used, as it is more suitable for parenteral pharmaceutical applications when compared to unsaturated monoglycerides (uMGs)-based cubic phase materials, which exhibit toxicity when injected in vivo at high concentration [75]. Cytryniak et al. reported the use of cubosomes for the first time as a dual-modality drug delivery system for internal radiotherapy combined with chemotherapy [76]. Cryo-TEM revealed a well-ordered structure inside these monoolein-based cubosomes loaded with doxorubicin and radionuclide 177. Angelov et al. identified extra-large nanochannels in cationic particles stabilized by PEGylation (Monoolein/dioctadecyldimethylammonium bromide (DOMA)/DOPE-PEG2000) [77]. Further studies by Angelov et al. also enabled the observation of small cubosome particles with well-defined water channels, precursors of larger cubosomes, which confirms the nanochannel-network growth in diamond-type cubic lipid particles [77].

Cryo-Field Emission Scanning Electron Microscopy (Cryo-FESEM) was used to obtain 3D structural information concerning cubosomes and hexosomes [78,79]. In contrast to Cryo-TEM, the Cryo-FESEM did not provide any information regarding the internal structure, but provided surface and overall morphology insights [72]. This technique allowed the description that the bulk cubic phase and cubosomes prepared from phytantriol and pluronic F127 exhibited a tortuous structure and bicontinuous nature with a non-intersecting network of water channels [79].

Recently, Demurtas et al. illustrated the power of Cryo-Electron Tomography (CET), which was used to uncover the surface structure between the cubic phase particle and the surrounding water. The resulting interpretation is that the interior constituted a perfect bicontinuous cubic phase, while the outside shows interlamellar attachments, which represent a transition state between the liquid crystalline interior phase and the outside vesicular structure [80].

###### 2.3.4.1.2. Small Angle X-ray Scattering (SAXS)

Scattering techniques have been vastly used in surface and colloidal science to investigate size, shape, and particle interactions in bulk solution, and also at interfaces [81]. Scattering techniques are non-invasive, which is an advantage, and some implementations allow their use in situ to characterize ongoing processes [82,83]. These techniques do not rely on the addition of probes or other additional molecules. The result represents an average of the physical properties of the sample.

SAXS is a powerful technique to identify the crystallographic structure of liquid crystalline phases [84]. It is by far the most frequently used method, and therefore we will limit our discussion to this technique.

In a SAXS experiment, X-rays interact with the electrons in the particles and are scattered elastically. SAXS identifies periodic spatial arrangements of different groups in any given direction.

It is worth highlighting that SAXS is commonly used to complement Cryo-TEM and other analytical methods for liquid crystalline structure verification. In this way, SAXS provides a global view of the ordered structure of the sample, while CryoTEM informs a local view on the cubosome structure at the individual particle level. According to Angelov et al., the structural SAXS data was used to confirm the formation of extra-large aqueous channel on for cubosomes formed by Monoolein/DOMA/DOPE-PEG2000 [77]. Rizwan et al. complemented Cryo-FESEM micrographs on cubosomes prepared with phytantriol and pluronic F127 with SAXS, confirming a bicontinuous cubic liquid crystalline phase with Pn3m geometry [79].

Structural SAXS data is also fundamental to characterize new cubosome formulations for diverse applications. Mathews et al. developed a cubosome for in vivo drug delivery applications. The system SAXS pattern revealed a hybrid cubosomal lipid nanocarriers with a pH-sensitive shell created by biopolymer complexes [85]. Johnsson et al. reported that the Bragg peaks identified in the X-ray diffractograms for a DOPE/PEG(660)-GMO/water system could be associated with an Ia3d cubic phase (45 wt% H_2_O) and the Pn3M cubic phase (50 wt% H_2_O) [75]. Ha et al. confirmed the internal crystalline structure of polymer cubosomes using SAXS. Most presented a Im3m or Pn3m geometry, however the gyroid (Ia3d) was also observable [86].

SAXS experiments have also been used to investigate cubosomes phase behavior in response to loaded molecules. A few examples include indomethacin [87], curcumin [44], capsaicin [88], rapamycin [89], Fe_3_O_4_ magnetic particles [90], cisplatin, and paclitaxel [91]. In addition, the effect of external parameters can also be analyzed using SAXS. Yang et al. used SAXS to show that the process of homogenization applied on a phytantriol-based cubosome dispersion did not lead to changes in the internal nanostructure [92].

Although SAXS is regarded as one of the most reliable structure determination methods, it is not void of limitations. Problems associated with SAXS include weak reflections when acquired using a lab source. This weakness is even more significant when dealing with dispersed liquid crystalline systems due to their small size and potentially non-uniform crystallographic microstructure [74]. Moreover, some systems may exhibit two or several co-existing mesophases, making the assignment of SAXS reflection peaks to specific groups a complex task [72,74].

##### 2.3.4.2. Indirect Techniques

###### 2.3.4.2.1. Dynamic Light Scattering (DLS) and Zeta(ζ)-Potential

DLS is one of the most ubiquitous methods to study particle size and dynamics in colloidal systems. It is a relatively simple non-invasive method to characterize particles in suspension. When a particle solution is irradiated with a monochromatic coherent light, the beam is scattered in all directions, creating a grainy speckle pattern. Particles in solution are not static. They undergo Brownian motion, and their local concentration fluctuates in time. In a DLS experiment, the fluctuations of the light intensity (*I*) of these speckles, due to the diffusion of particles with time (*t*), are recorded and translated into an autocorrelation function, *g*^2^(*t*) (6):

(6) $$g^{2}\left(t\right)=\frac{I\left(t\right)I\left(\;t+\tau \right)}{I{\left(t\right)}^{2}}$$

where *I*(*t*) is the intensity of the scattered light at a time (*t*) and *I*(*t* + *τ*) the light scattered at time (*t* + *τ*), respectively [93,94].

This technique is often used to measure the size of the prepared cubosomes.

The major drawback of DLS measurements is the fact that heavier and larger particles contribute more strongly to the overall mean decay rate of a polydisperse solution, which often lead to an overestimation of such larger particles. Despite this, this valuable information relative to translational diffusion of the particles, and therefore particle size, runs hand in hand with characterizing cubosomes. The sizes of “honeycombed” structured cubosomes have been measured for a large variety of different systems destined for various applications, ranging from 10–500 nm in diameter [49].

Malheiros et al. used the DLS technique to analyze the average hydrodynamic diameter and polydispersity of phytantriol cubosomes prepared in full hydrated conditions in the presence of increasing hexadecylphosphocholine (HePC) concentrations [95]. They demonstrated that there was an increase of 2-fold in the polydispersity index at both 10 and 14 mol%, revealing the effect of the HePC in the colloidal system at these higher concentrations [95]. In turn, Victorelli et al. developed mucoadhesive cubosomes for the delivery of curcumin for cervical cancer treatment and used DLS to characterize the system. The curcumin-loaded cubosomes exhibited a homogeneous size distribution with a polydispersity index lower than 0.4 [96].

Another important parameter and often accessed for liquid crystalline systems is the determination of ζ-potential [76,88]. ζ-potential measurements can only be performed indirectly, where experiments are designed to measure the velocity of a charged particle that moves under the influence of an applied electric field (electrophoretic mobility). For pharmaceutical applications, ζ-potential is crucial to determine the existence of cationic or anionic particles in solution. For instance, Victorelli et al. was able to confirm the presence of positive charges on curcumin-loaded cubosomes through ζ-potential measurements [96]. Patil et al. developed an inhalable bedaquiline-loaded cubosome nanocarriers for non-small cell lung cancer treatment. The ζ-potential measurements showed that the nanocarriers were cationic and stable regarding particle size for periods of eight weeks at storage conditions [97].

###### 2.3.4.2.2. Nuclear Magnetic Resonance (NMR)

NMR is a multifaceted technique which can be used to determine physical and chemical properties of atoms and molecules, providing detailed information in terms of structure, dynamics, reaction state, and chemical environment of the molecules. It is worth highlighting the pioneering work of Pieter Cullis when discussing the use of NMR to characterize biological and synthetic lipidic systems [98,99,100,101,102,103,104,105,106,107,108].

Rajesh et al. explored lipidic poly(2-methyl-2-oxazoline) (PMeOx) as an alternative to F-127 to stabilize cubosomes. Two lipopolymers, PMeOx40-OA and PMeOx80-OA, with different degrees of polymerization, were successfully synthesized and analyzed for end-group efficiency by 1H NMR. The 1H-NMR pattern showed that polymers had a very high degree of end-group functionalization (≥95%), therefore suitable for the preparation of PMeOx stabilized cubosomes [109].

###### 2.3.4.2.3. Rheology

Rheology deals with how a system responds to mechanical perturbation in terms of elastic deformations and of viscous flow [110]. Shear rheology can provide both structural and dynamical insights of liquid crystalline mesophases [72]. Viscoelastic materials are usually independent of strain up to a critical strain level. The viscoelastic behavior of samples is investigated by measuring the strain amplitude dependence of the storage and loss moduli (G’ and G”, respectively).

The general behavior for cubic phases of G′ and G” as a function of frequency is G″ predominates at lower frequencies, while G′ is dominant at higher frequencies [111]. G′ > G″ indicates that the material is highly structured and behaves solid-like. Increasing the strain above the critical strain disrupts the network structure and the material becomes progressively more fluid-like (G′ < G″). The frequency at which the crossover, G′ > G″, occurs depends on the composition of the sample [111].

Among the liquid crystalline phases, bicontinuous cubic phases (Ia3d, Pn3m, and Im3m) are regarded as the most rigid structures followed by the reverse hexagonal phase, which possess intermediate viscoelastic properties, and the lamellar phase, which is characterized by plastic fluid properties [112].

Mezzenga et al. reported that each distinct liquid crystalline phase has a specific rheological signature. The measurements of G’ and G” as a function of temperature and composition can also reveal structural transitions between liquid crystalline structures [112,113,114]. Based on these differences in viscoelastic behavior, structural transitions between the mesophases have been detected in several experimental systems, including cubic-to-hexagonal, cubic-to-cubic, and hexagonal-to-isotropic fluid [112,113,115,116,117].

It was also shown that cubic phases have different characteristic relaxation times [113]. The relaxation time of the systems is obtained from the inverse of the frequency at which the crossover of G’ and G’’ takes place [112]. It can be viewed as the time scale for the water–lipid interface to relax to or obtain equilibrium configuration, after having been perturbed by shear deformations [111,114].

Villalva et al. immobilized cubosomes in oxi-HA/ADH hydrogels and performed rheological studies demonstrating that the presence of both anionic and cationic cubosomes slightly increased the G’ values for hydrogels of low and high degrees of cross-linking with respect to their respective pure hydrogels. Therefore, the cubosome charge does not affect the mechanical properties of the hydrogel [118]. These rheological properties confirmed that the *in situ* applicability of the hydrogel is not affected.

It is worth mentioning here that, in recent years, Fluorescence Recovery After Photobleaching (FRAP) has become a common tool to evaluate lipid self-diffusion [119,120,121]. FRAP is a fluorescence-based technique which evaluates diffusion of a fluorescent material into a region, where a high intensity laser has previously produced photobleaching.

###### 2.3.4.2.4. Polarized Light Microscopy

The addition of cross polarizers can enable distinctions between phases that, to the naked eye, may seem identical. This means that polarized light microscopy can reveal the morphology of the liquid crystalline based on the optical birefringence phenomena, distinguishing between anisotropic and isotropic arrangements [122,123]. For example, hexagonal liquid crystalline due to the anisotropic molecular arrangement will appear to be shinning when observed through crossed polarized light due to birefringence. For cubic liquid crystalline, the isotropic molecular arrangement is non-birefringence and therefore will display a dark field in the polarizing image [124,125]. Among all the techniques, polarized light microcopy provides the easiest way to qualitatively distinguish between phases.

Tian et al. developed folate-modified cubosomes containing etoposide in order to achieve better targeting properties and therapeutic effects compared with the traditional cubosomes. The prepared cubosomes were characterized by polarized light microscopy, which confirmed their cubic morphology [126]. Additionally, Fan et al. developed a liquid taste-masking system based on lyotropic liquid crystalline nanoparticles (LLCNs) for pediatric medicine [127]. The authors also encapsulated cefpodoxime proxetil (CFP) into the LLCNs (CFP- LLCNs) to improve their taste and thus facilitate the administration of drugs to children. The mesophase analysis of the prepared nanoparticles was performed by polarized light microscopy and SAXS, revealing the cubic phase of CFP- LLCNs and blank LLCNs, therefore confirming that CFP loading did not influence the phase of LLCNs [127].

###### 2.3.4.2.5. Differential Scanning Calorimetry (DSC)

DSC is extremely valuable to study the thermotropic behavior of diverse systems due to the extractable information in terms of transition temperatures and enthalpies of transitions. Liquid crystalline is a thermodynamic equilibrium system. A phase transition is usually accompanied by endothermic or exothermic energy changes, which can be determined by DSC [122].

Mansour et al. prepared dexamethasone-loaded cubosomes that were subjected to DSC studies in order to detect the effect of excipients and process parameters on the physical stability of the formed particles. These studies showed that the typical endothermic peak of dexamethasone disappeared in the medicated lyophilized cubosomes, suggesting its entrapment into the formed cubosomes in an amorphous form [128]. Furthermore, Zhang et al. loaded anticancer drugs, namely, cisplatin and paclitaxel in cubosomes coated with a layer of poly-ε-lysine. The prepared cubosomes were subjected to DSC studies, which indicated that the drugs were not present in the cubosomes in crystalline form but dispersed all throughout the cubosomes [91].

###### 2.3.4.2.6. Entrapment Efficiency

Cubosomes have endless potential as drug delivery systems as these systems can retain adequate amounts of small drugs, peptides, biologic, or bioactive molecules. There are numerous approaches for loading the drug into cubosomes, with a common denominator of success reported as the entrapment efficiency. Entrapment efficiency and drug loading of cubosomes can be accessed resorting to chromatography techniques [76], dialysis [88], small-angle X-ray scattering [37], or ultra-filtration techniques [89]. The amount of unentrapped drug can be further analyzed using a UV spectrophotometer [44,89], fluorescence [44], HPLC analysis [88,123], Fluorescence Correlation Spectroscopy [90], or radioactivity [76] for the purpose of, for example, obtaining a drug release profile.

Alharbi et al. developed ciprofloxacin-cubosomal (Cf-Cub) in situ gel with the aim of improving eye permeation, prolonging the ocular retention time, and enhancing its antimicrobial activity. In this study the entrapment efficiency was determined, showing its dependence on factor levels in formulations [129]. Moreover, Elakkad et al. developed transcutaneous tenoxicam loaded hyalcubosomes—prepared by adding sodium hyaluronate to cubosomes components—to control osteoarthritis without common side effects [130]. The entrapment efficiency percentage of the developed vesicles was determined by indirect technique. This study revealed that the entrapment efficiency of the tenoxicam-loaded hyalcubosomes is slightly higher, at around 3%, when compared to tenoxicam-loaded cubosomes [130]. These results may be due to the rigidly structured vesicle formed by hyaluronate, which favored the inclusion of tenoxicam inside the vesicles.

###### 2.3.4.2.7. Stability Studies

The physical stability of cubosomes is studied by the investigation of organoleptic and morphological aspects as a function of time. Essentially, all methods described above can be used in a manner to evaluate these aspects. The verification of the well-defined inner structure characteristic of cubosomes can be performed at different time-points. Particles size distribution and drug content can be assessed over time and be used to evaluate possible variations.

As previously mentioned, Elakkad et al. prepared tenoxicam-loaded hyalcubosomes and tenoxicam-loaded cubosomes. These samples were also subjected to a stability study, which showed the particle size, zeta-potential, and entrapment efficiency of cubosomes formulation and hyalcubosomes formulation before and after 3 months at 4 °C ± 1 [130]. This study allowed us to conclude that storage of the prepared vesicles in tightly closed amber glass containers at temperature 4 °C did not adversely affect either the particle size and PDI, while zeta potential and entrapment efficiency percentage were affected [130]. In addition, Bessone et al. developed Latanoprost-loaded phytantriol cubosomes for the treatment of glaucoma [131]. The prepared samples were stored in tightly closed amber glass vials in a controlled humidity thermostatic cabin (25 °C) for a period of 30 days. In this way, the stability studies demonstrated that the concentration of latanoprost in the cubosomes and the physicochemical properties did not change over one month, evidencing good stability [131].

## 3. Medical Applications of Cubosomes

The characteristics of cubosomes make them promising solutions to several biomedical applications. The ability to incorporate hydrophobic, hydrophilic, and amphiphilic compounds, the capacity to protect and safe deliver molecules, and the tortuous internal configuration that allows a sustained release are a few examples of features that make cubosomes particles with such great potential. From therapeutics to imaging, or even a combination of both, innumerable applications have been proposed regarding the use of cubosomes in the biomedical field, such as in cancer [132], infection [133], or even cerebrovascular disorders [64]. In this review, some examples are presented.

### 3.1. Cancer Therapy

Cancer is a major global health challenge, with millions of new cases every year, and represents the second leading cause of death according to World Health Organization. The scientific evolution allows the discovery of new approaches towards the treatment of different types of cancer. In the development of new strategies, and specifically new drugs, some of the challenges are the administration, the transport, and stability of the compound inside the organism and the ability to arouse the wanted effect. Nanocarriers play an important role in overcoming these obstacles, as their main purpose is to deliver the molecules of interest safely and efficiently to the desired location. A good drug delivery system allows the reduction of the drug dosage without compromising the therapeutics, and subsequently diminishes the side effects. Several studies have been published regarding the application of cubosomes as a nanocarrier for cancer therapeutics. Doxorubicin [76], cisplatin [91], paclitaxel [91,134], curcumin [96], and quercetin [135] are a few examples of molecules that have been successfully encapsulated in cubosomes, either alone or in some kind of simultaneous encapsulation.

Zhai et al. studied the efficacy of monoolein cubosomes loaded with paclitaxel in the treatment of ovarian cancer [57]. The in vitro results in HEY cells (HX-62) showed that the blank cubosomes did not present cytotoxicity, and that loaded cubosomes were effective in the tumor inhibition, possessing a more efficient action than free paclitaxel. In vivo experiments proved the previous results and allowed us to conclude that the loaded cubosomes increased the survival of the tumor bearing mice.

In another work, L. Zhang et al. attempt to simultaneously encapsulate two different compounds into the cubosomes. Monoolein cubosomes were loaded with either cisplatin, paclitaxel, or a combination of both, and coated with poly-Ɛ-lysine. Cytotoxicity and action against cancer cells were evaluated by MTT assay on human hepatoma Hep G2 cells and fluorescence on HeLa cells, respectively. The obtained results showed that the coated cubosomes had a higher encapsulation efficiency, significantly reduced the initial burst release, and allowed for a controlled and slow release of the molecules. Furthermore, the coating allowed for an increased viability of the Hep G2 cells and a death increase on HeLa cells. Finally, the cubosomes with a combination of paclitaxel and cisplatin showed a higher efficiency against HeLa cells when compared to single-loaded cubosomes [91]. Such results evidence that cubosomes have huge potential as drug carriers and may be a solution to reduce the drug dosage without compromising the therapy.

Bazylinska et al. developed a photodynamic approach towards the treatment of skin malignant melanoma. Monoolein cubosomes were loaded with Chlorin e6 (Ce6) or meso-Tetraphenylporphine-Mn (III) chloride (TPP-Mn), two photosensitizing dyes. The cubossomal solution showed particles with an average size of 130 nm and low polydispersity (0.13). In vivo experiments regarding cellular uptake, cytocompatibility, and photodynamic activity were conducted in two human malignant melanoma cell lines (Me45 and MeWo). In both cases, the uptake of the encapsulated photosensitizers was greater when compared to the free form of these compounds. Furthermore, Ce6-loaded cubosomes displayed low cytotoxicity prior to irradiation, and high cytotoxicity after the energetic input, therefore being biocompatible therapeutic-effective particles. In the case of TPP-Mn, a low photocytotoxicity was obtained. The authors conclude that despite this downside, TPP-Mn’s high cellular uptake could be used for cancer cells imaging [136].

The unique characteristics of cubosomes makes them interesting to use in new cancer therapy approaches. An innovative work studied the use of cubosomes as simultaneous carriers of doxorubicin, a chemotherapeutic, and Lutetium-177, a radionuclide, thus allowing for a combined chemo- and radiotherapy [76]. In the experiment, the combination resulted in stable cubosomes, but with insignificant cancer therapeutics when compared to other solutions. The authors conclude that the doxorubicin presented a much higher cytotoxicity than Lutetium-177, therefore further studies are needed, and with a different type of radionuclides that should be able to deposit more energy in shorter periods [76].

**Figure 9:**
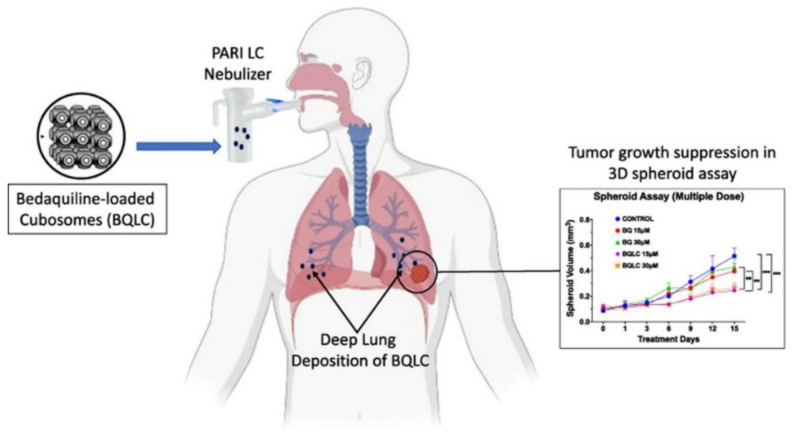
Conceptual representation of the work by Patil et al., 2021. Bedaquiline-loaded cubosomes were designed for inhalatory administration in the context of cancer therapy. Reproduced with permission from reference [97]. Copyright 2022 Elsevier.

Additionally, it is worth noticing the development of inhalable bedaquiline-loaded cubosomes (BQLC) for non-small cell lung cancer treatment (NSCLC) (Figure 9) [97]. These nanoparticles were prepared via a solvent evaporation technique, showing excellent characteristics such as particle size (150 nm), zeta potential (35 mV), and aerosolization behavior. This study evidenced the efficiency of the nanoencapsulation of bedaquiline, an anti-tuberculosis drug with great anti-cancer efficacy, allowing to overcome its poor water solubility that limits its delivery through the lungs. Indeed, the successful internalization of the BQLC in lung cancer cells (A549 cells) has been revealed, which resulted in the decrease of the half-maximal inhibitory concentration (IC50) (3-fold), as well as in the significant inhibition of tumor growth compared to free bedaquiline. The excellent aerosolization performance and improved anti-cancer activity demonstrated by the obtained results highlight the potential of BQLC as NSCLC therapy [97].

### 3.2. Transfection

Gene therapy consists of the use of nucleic acids to achieve a genetic manipulation of specific cells. It can be used, for example, in the treatment of monogenic diseases by substituting the missing or mutated gene with a normal allele [137]. In addition, gene therapy can be used in the treatment of cancer, AIDS, Parkinson, among others [138]. Despite its potential, gene therapy use has been limited due to the lack of efficient carriers. This difficulty is related with finding carriers that fulfil the requirements needed, for example: avoid the degradation of the nucleic acids, facilitate cellular uptake, and promote the release of the genetic molecule in the cell [139]. In the case of viral vectors, these carriers have shown immunogenicity and are difficult to handle and to produce on a large-scale. In terms of physical gene transfection systems, some of its disadvantages are the difficult large-scale production and the low transfection efficiency. Chemical carriers have been created with the aim of overcoming the drawbacks of viral and physical systems. Lipids have the ability to interact with plasmid DNA and consequently pass the cell membrane, therefore being one of the materials used in the development of such systems [140]. Cubosomes are lipidic nanoparticles, hence they can be a good solution for the delivery of the genetic molecules.

To explore the differences between the use of cubosomes and liposome-based systems as carriers of siRNA, Kim et al. developed “PEGylated cuboplexes”: monoolein and DOTAP cubosomes coated with PEG and loaded with siRNA. In their work, HeLa cells were pre-treated to express firefly luciferase (FF) [71]. Afterwards, cubosomes targeting the FF mRNA for sequence-specific degradation were added to the cells. As a result, the cuboplexes successfully delivered siRNA to the cells and silenced luciferase. Furthermore, the developed particles showed a better efficiency when compared to the classical liposomal alternatives. In another work, Kim et al. developed cuboplexes using microfluidics [141]. This method allowed to achieve a better control over the particles size and polydispersity without compromising the ability of the particles to carry siRNA, consequently promoting the desired gene-silencing.

**Figure 10:**
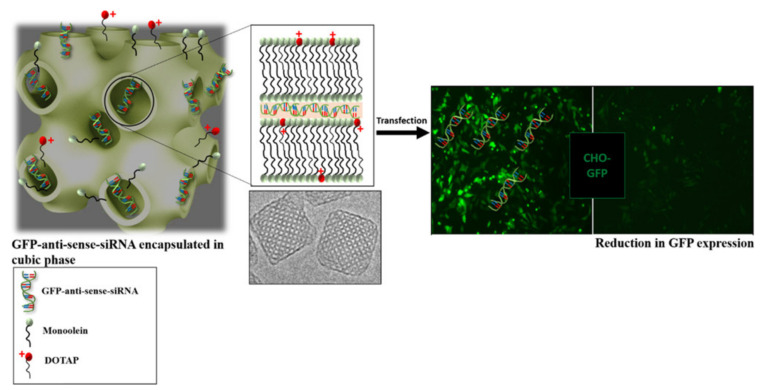
Conceptual representation of the work by Sarkar et al., 2021. Cubosomes loaded with GFP-antisense siRNA are shown to reduce the expression of GFP in vitro using Chinese Hamster Ovary cells. Reproduced with permission from reference [139], 2022.

Another example of a successful transfection using cubosomes was reported by Sarkar et al. (Figure 10). Different combinations of monoolein doped with cationic lipids were used in the assembly of cubosomes loaded with antisense green fluorescent protein (GFP). GFP expressing Chinese Hamster Ovary cells were treated with the cubosomes. The results allowed us to conclude that the knockdown efficiency is related to the cationic lipid used and that, for a period of up to 48 h, some of the combinations exhibit a gene knockdown efficiency similar to lipofectamine (liposomal-based formulation that is commercially available), and a higher efficiency after 72 h [139].

Gajda et al. made an interesting therapeutic combination where monoolein cubosomes were loaded with miR-7-5p (miRNA) and doxorubicin (DOX- chemotherapeutic) for a glioblastoma treatment [142]. Drug-sensitive (A172 and TPC-1) and drug-resistant (HeLa and T98G) cell lines were used to test the efficacy of the combined treatment with the cubosomes loaded with miRNA and DOX. The results showed that loaded cubosomes with DOX/miR-7-5p were more effective in inducing cellular apoptosis than cubosomes loaded only with DOX. The authors conclude that miR-7-5p allows the reduction of cells’ drug-resistance, thereby enhancing the efficiency of the drug.

### 3.3. Topical Drug Delivery and Antimicrobial Therapy

Transdermal drug delivery allows for a pain-free drug delivery that can avoid first pass metabolism, thus allowing a lower dosage. However, there are some difficulties in the use of this administration route. One example is the stratum corneum, one of several layers that compose the skin, which acts like a barrier, making it difficult for the molecules to penetrate [49]. Lipid-based colloidal systems can help to fluidize skin lipids, allowing to enhance the penetration of molecules. Cubosomes can, therefore, be a possible vehicle in the delivery and transport of drugs to and through the skin. Moreover, cubosomes also have great potential to be used in antimicrobial therapy, enhancing the action of drugs against pathogens.

Boge et al. explored the ability of cubosomes to topically deliver LL-37, an antimicrobial peptide (AMP) [143]. LL-37 was loaded into monoolein cubosomes by three different approaches: pre-loading, post-loading, and hydrotrope-loading, which correspond to the encapsulation of the compound prior, after, and during the cubosomes formation, respectively. Results showed that the encapsulation approach had an impact on the cubosomes sizes and structures, with hydrotrope-loading showing the smallest cubosomes. Neither of the formulations evidenced skin-irritation. Under physiological conditions, some AMPs are susceptible to proteolytic degradation. Once exposed to Pseudomonas aeruginosa elastase or human neutrophil elastase, pure LL-37 was completely degraded, whilst the LL-37 loaded into the cubosomes remained intact. Furthermore, the encapsulated LL-37 displayed bactericidal effects against E. coli and S. aureus, even after the enzyme exposure [143].

Bioadhesion of cubosomes makes them an appropriate choice as carriers for the delivery of molecules to external tissues. Monoolein cubosomes were loaded with norfloxacin, an antibiotic used in the treatment of acute otitis externa, usually caused by bacteria, mainly pseudomonas. In vivo tests conducted in rabbits evidenced that the use of cubosomes as carriers allowed to improve the deposition of norfloxacin in rabbit ears, in comparison with a non-encapsulated norfloxacin suspension. This confirmed the ex vivo study results, which also showed a better penetration of the encapsulated norfloxacin. Histopathological examinations did not reveal any signs of skin irritation. This demonstrates the potential use of cubosomes as carriers for the enhancement of transdermal delivery of norfloxacin [144]. These results are highly interesting and can enhance the topical application of other types of molecules (natural and synthetic), modernizing the dermocosmetic industry or even medicine.

Glaucoma is a major global health problem that can lead to irreversible blindness. In a glaucoma emergency, acetazolamide is the drug of choice. However, the only possible administration is via systemic tablets. Teba et al. proposed the formulation of a cubosome-based system for topical delivery of acetazolamide [145]. In their work, the results allowed the conclusion that the optimized formulation of acetazolamide-loaded monoolein cubosomes were not an irritant for the eye and were able to arouse a longer lasting and effective therapeutics than other pharmacological solutions commercially available. In addition, Bessone et al. prepared latanoprost-loaded phytantriol cubosomes (CubLnp) using a top-down method [131]. These cubosomes showed a very slow release of Latanoprost, an anti-glaucoma drug, evidencing a sustained release profile. It has also been shown that a single subconjunctival application of CubLnp induced greater reduction in intraocular pressure of around 30% at 24 h. Furthermore, low concentrations of CubLnp revealed a hypotensive effect, with an intraocular pressure decrease of 20%.

Cubosomes can also be incorporated in hydrogels in order to obtain a better sustained drug delivery system [118,146,147]. In this way, Sanjana et al. developed and characterized a novel cubosomal formulation, including dexamethasone (DMS), for vitiligo treatment [146]. The cubosomes were prepared through top-down technique and were incorporated into hydrogel for prolonged delivery of DMS. The cubogel was formed by incorporating an optimized formulation in 1% carbopol 940. The prepared cubogel exhibits excellent properties, such as enhanced spreadability, higher drug content, ideal pH, and a beneficial sustained release pattern at the end of 12 h, that makes it suitable to be a promising topical sustained drug delivery system to treat vitiligo. Moreover, Rapalli et al. developed topical hydrogel consisting of ketoconazole-loaded cubosomes with lower surfactant concentrations [147]. This hydrogel showed a higher permeation and skin retention, as well as an enhanced anti-fungal activity, being an interesting strategy for topical drug delivery. Villalva et al. successfully immobilized diclofenac-loaded cubosomes in oxi-hyaluronic acid/ADH hydrogels, revealing its potential as an important platform for biomedical applications in drug delivery [118].

Recently, Lai et al. showed that polymyxin-loaded cubosomes could disrupt the outer membrane of Gram-negative bacteria, reaching a superior polytherapy activity [148]. Indeed, the outer membrane was initially destabilized by the electrostatic interactions between polymyxin and lipid A, and subsequently, the membrane was further disrupted through a lipid exchange process caused by an influx of cubosomes.

### 3.4. Vaccines

Vaccination is an essential way to protect the body from bacterial or viral infections and cancer. An effective vaccine should motivate strong humoral and cellular immune responses. The success of vaccines involves antigen and matching adjuvants. So far, a variety of nanosystems have been developed into adjuvants, such as liposome, PLGA, and cubosomes. In specific cases of cubosomes, recent studies have revealed their adjuvant activity and potential utility as an antigen delivery system. The recent investigations found that cubosomes have the ability to promote more humoral and cellular immune responses than antigen alone.

Ginseng stem–leaf saponins (GSLS) present excellent immunoregulatory applications, but a low bioavailability. Qiu et al. studied chitosan modified monoolein cubosomes, prepared by solvent shifting, and loaded with GSLS and ovalbumin (OVA-antigen). The obtained cubosomes did not display cytotoxicity and an increase in the uptake efficiency of OVA was registered. Furthermore, the results exhibited a high production of OVA-specific IgG antibodies, and a better cellular immune response compared to the other studied groups (e.g., cubosomes loaded with OVA, but not with the GSLS). In vivo results showed that the particles could improve lymphocyte activation and immune response in splenocytes [149].

Thereby, the cubosomes demonstrate great potential to be used as carriers for transport and targeted delivery of antigens and vaccines adjuvants.

### 3.5. Imaging and Theranostic

Medical imaging represents an important tool in the correct diagnosis of diseases. It can also play a major role in treatments by providing a picture of the therapy’s impact. There are several techniques that can be used according to each situation, such as X-rays, Magnetic Resonance Imaging (MRI), or Ultrasounds. Some of these techniques, like the MRI, require the injection of specific agents into the patient’s body for visualization. Cubosomes small size, biocompatibility, and low viscosity make these nanoparticles appropriate for intravenous administration. In addition, their ability to carry different types of molecules allow cubosomes to be employed in tissue targeting and multimodal images.

Super magnetic iron oxides nanoparticles (SPIONs) are MRI contrast agents that act by influencing the longitudinal relaxation time (T1) and transverse relaxation time (T2). Phytantriol cubosomes stabilized with Pluronic F-127 were loaded with SPIONs. The magnetic nanoparticles were successfully incorporated and did not affect the cubosome structure. Then, live rats were injected intravenously with the prepared particles, and scans of the liver and kidneys were obtained before and after the application of the cubosomes. Comparing to monodisperse SPIONs, the SPIONs loaded cubosomes achieved an improvement in the contrast of T2 weighted images of the liver and kidneys of the rats. Therefore, cubosomes were efficient in the transport and subsequent delivery of an MRI contrast agent [150].

In a theranostic approach, both drugs and imaging agents are simultaneously transported and delivered to the targeted site by the same carrier. Murgia S. et al. [135] studied the potential of cubosomes as carriers for a theranostic application. Monoolein cubosomes were firstly decorated with two hydrophobically modified fluorophores (fluorescein, F1 and dansyl, F2). While both probes were successfully incorporated, a cryo-TEM analysis showed that F2 induced an inner structural change. Then, F1-doped cubosomes were incubated with 3T3 fibroblasts cells. After the incubation period, a diffuse fluorescence was observed in the cytosol, especially in the perinuclear region, thus proving that cubosomes can act as a carrier for imaging agents. Quercetin was then loaded into the cubosomes, resulting in an approximate loading-efficiency of 80%. The particles did not suffer any significant structural changes. In a similar work, Caltagirone et al. were also successful in loading monoolein cubosomes with an NIR-emitting fluorescent probe and camptothecin, an anticancer drug [151]. Bazylińska et al. also studied the potential of hybrid cubosomes towards a theranostic approach [152]. Daunorubicin was loaded into monoolein cubosomes in conjugation with lanthanide-ion doped fluoride up-converting nanoparticles. The formed particles successfully underwent cellular uptake (KOV-3 and MeWo cancer cell lines) and exhibited photocytotoxicity. Such results evidence the cubosomes’ potential as a theranostic carrier.

## 4. Concluding Remarks

The development of nanocarriers led to important advances in several research areas, particularly in the medical field. Research on nanocarriers has proven to increase the efficiency of medical treatments, mostly due to their ability to target specific cells. Among all the nanocarriers developed and characterized, cubosomes have been drawing great attention in recent years. In this article, we have reviewed the main physicochemical concepts and parameters ruling cubosome assembly, as well as the most common characterization techniques reported and some of the biomedical applications for which cubosomes have been proposed. The main interest behind the biomedical use of cubosomes relies on their ability to incorporate molecules with different physicochemical properties, such as polar and apolar molecules. This property enhances the efficiency of a treatment as it allows the synergy between two molecules, which will be delivered simultaneously. Moreover, the versatility of cubosomes does not cease on the ability to encapsulate more than one molecule simultaneously, but it has been successfully used as a theranostic tool. Cubosome development has open the door to new and more efficient therapies with less secondary effects. It is envisaged as an improvement on cubosomes’ preparation methods, leading to even more applications.

## Data Availability

Not applicable.
